# The Novel Role of Healing from Bacterial Infections of Lower Limb Open Fractures by X-Ray Exposure

**DOI:** 10.1155/2020/3129356

**Published:** 2020-03-19

**Authors:** Ali A. Mahdi, Tuqa S. Al-Salmani, Mustafa M. Al-Qaisi

**Affiliations:** College of Health & Medical Technology, Middle Technical University, Baghdad, Iraq

## Abstract

**Objective:**

To confirm the role of X-ray exposure in treating infected wound fractures at the lower limb and determine X-ray exposure times.

**Methods:**

Fifty-one wound swabs were collected from patients with infected open fractures at the lower limb with grade II, IIIA, B, and C according to the Gustilo and Anderson classification system and then cultured. The bacterial isolates were identified by biochemical tests and the VITEK-2 System and tested against several antibiotics. The X-ray exposure was done for open fractures by radiography (at kV133 and 5 milliambers).

**Results:**

The higher isolation rate was recorded for *Staphylococcus aureus* with 21 (41.2%) isolates, and most of them (20, 95.2%) were isolated from grade II fractures. The isolation rate of Gram-negative bacteria was 25.5% for *Escherichia coli* with 13 isolates, 19.6% for *Pseudomonas aeruginosa* with 10 isolates, and 13.7% for *Klebsiella pneumoniae* with 7 isolates, most of which were isolated from grade III fractures. The isolation rate of *P. aeruginosa* was 60% (6 isolates) from grade IIIA and 71.4% (5 isolates) from grade IIIB for *K. pneumoniae*, while for *E. coli* it was 69.2% (9 isolates) from grade IIIC. All the bacterial isolates recorded high levels of antibiotic resistance against most tested antibiotics. Wound cultures of grade II fractures appeared sterile after the first X-ray exposure, and these wounds were infected with *S. aureus* or *P. aeruginosa*. However, cultures of grade IIIA and IIIB fractures appeared sterile after the second X-ray exposure for all isolated bacteria, except for *S. aureus* (grade IIIA fractures) (after the third X-ray exposure). Grade IIIC fractures showed sterile culture after the third X-ray exposure for wounds infected with *P. aeruginosa* and *E. coli*.

**Conclusions:**

The study concluded that X-ray exposure showed high effectiveness in treating infected open fractures.

## 1. Introduction

An open fracture refers to one type of injury where there is a break in the skin and soft tissue, resulting in the exposure of the fractured bone to the external environment [1], leading to an increase in the eventuality of microbial contamination. The infection subsequently assures with serious complications, and treating open fractures has been very difficult [2]. The open fracture treatment differences depend on the type of fracture and severity of the injury, while the treating management includes aggressive irrigation of these wounds, early surgical debridement, administration of antimicrobial agents, and fixation of the fracture [3].

Open fractures are the most common cause of morbidity and mortality following traumatic injuries, and the infections remain the most complicated problem among these wounds [4]. Yearly, the incidence of open fractures recorded as 11.5 per 100 000 persons and >70% involve the lower limb [5]. The severity of open fractures was classified according to the Gustilo and Anderson classification as grades I, II, and III, and grade III was subclassified into IIIA, IIIB, and IIIC depending on the extent of contamination and the degree of soft tissue damage [6]. Hazard infection is 0%–7% for grade I, 0%–11% for grade II, 2%–36% for grade III, and up to 44% for the grade IIIC subtype [7, 8]. The problem that accompanies open fractures is the infection; therefore, an important goal in open fractures treatment is preventing infection [9]. The first application of antibiotics in the treatment of open fractures started during World War II by using penicillin [10]. Antibiotics are now routinely administered as part of the trauma management protocol for open fractures, leading to an increase in the antimicrobial resistance of open wound fractures [11, 12]. Eventually, treating open fractures became very difficult, and choosing another role in treating became necessary.

X-rays are one type of ionizing radiation that has a penetrating ability to most tissues and kill bacteria by causing irreversible damage to DNA. This radiation can effectively kill many types of Gram-negative bacteria such as *E. coli*, *P. aeruginosa*, and *Salmonella* species [13–15]. The present study aims at proving the role of X-ray exposure in the healing of infected open fractures and determining the optimum number of X-ray exposure for healing from different bacterial causative agents.

## 2. Materials and Methods

### 2.1. Study Design

The study design of the present study is the experimental research design. The sample size of the study was not calculated due to the limitation of the research duration. The figures throughout the study were drawn using Microsoft Excel 2010. The outcomes of the study were analyzed statistically using SPSS (version 23) by using Fisher's exact test.

### 2.2. Sample Collection

Fifty-one wound swabs were collected from patients with severe inflamed open fractures following fixation management. These wound swabs were cultured on blood agar and MacConkey agar media. The severity of these wound fractures was classified into grade II and grade IIIA, B, and C according to the Gustilo and Anderson classification [6]. They were located at the lower limb of patients admitted (Dowaly Private Hospital) during the period from February 2017 to September 2018.

### 2.3. Bacterial Identification

All collected wound swabs were cultured on blood agar and MacConkey agar for screening. Whole bacterial isolates were identified to species level by the different standard microbiological and biochemical tests [16], and VITEK-2 System was used to confirm the identification.

### 2.4. Antibiotic Susceptibility Test

The disk diffusion method was utilized to detect the susceptibility pattern of bacterial isolates against antibiotics. The tested antibiotics can be classified into several classes as follows: (I) aminoglycosides: amikacin (AK) 30 *µ*g and gentamicin (CN) 10 *µ*g, (II) penicillins: ampicillin (AM) 10 *µ*g and oxacillin (OX) 5 *µ*g, (III) penicillin combinations: amoxicillin/clavulanic acid (AMC) 20/10 *μ*g, (IV) cephalosporins: second-generation drugs including cefoxitin (FOX) 30 *µ*g, third-generation drugs including ceftriaxone (CTR) 30 *µ*g and ceftazidime (CAZ) 30 *µ*g, and fourth-generation drugs including cefepime (CPM) 30 *µ*g, (V) carbapenems: imipenem (IPM) 10 *µ*g and ertapenem (ETP) 10 *µ*g, (VI) fluoroquinolones: ciprofloxacin (CIP) 5 *µ*g and levofloxacin (LVX) 5 *µ*g, (VII) sulfonamides: trimethoprim/sulfamethoxazole (SXT) 25 *µ*g, as categorized by Hassan et al. [17], (VIII) macrolides: erythromycin (E) 15 *µ*g, (IX) lincosamide: clindamycin (CLI) 2 *µ*g, (X) glycopeptides: vancomycin (VAN) 30 *µ*g, (XI) lipopeptide: daptomycin (DAP) 30 *µ*g, (XII) monobactams: aztreonam (AZT) 30 *µ*g, and (XIII) drugs against mycobacteria: rifampin (RIF) 5 *µ*g. The antimicrobial resistance of *S. aureus* isolates was examined against erythromycin, oxacillin, cefoxitin, clindamycin, daptomycin, vancomycin, and rifampin, while that of *P. aeruginosa* isolates was tested against ceftazidime, gentamicin, amikacin, aztreonam, cefepime, ciprofloxacin, levofloxacin, and imipenem. The Enterobacteriaceae including *E. coli* and *K. pneumoniae* were tested for ampicillin, amoxicillin/clavulanic acid, gentamicin, ciprofloxacin, levofloxacin, ceftriaxone, ertapenem, imipenem, and trimethoprim/sulfamethoxazole, as recommended by the CLSI (2014) [18].

### 2.5. X-Ray Exposure

All the studied patients were exposed to X-ray by radiography (at kV133 and 5 milliambers) for monitoring the healing of the fracture. The duration of exposure was the same duration of routine X-ray examination (approximately 15–30 ms). The number of X-ray exposure was one, two, and three times. The three exposures were enough to kill all isolated bacteria in the current study. The interval between each exposure was ten days. After each one of exposure, wound swab culture was repeated for screening for bacterial growth. The timing of the wound swab was noted after the second day of X-ray exposure.

## 3. Results

### 3.1. Demographical Data

Throughout the study, fifty-one patients suffering from infected open fractures were admitted into the hospital. The age of patients was ranged from 20 to 46 years with mean and standard deviation of 31.7 ± 5.97 years. According to gender, 29 (56.9%) patients were male and 22 (43.1%) patients were female. All studied fractures were located on the lower limb with severity grades of II, IIIA, IIIB, and IIIC according to the Gustilo and Anderson classification. All the studied patients were sent for culture, and all of them (51, 100%) showed bacterial growth. The open fractures have become sterile, and no growth appeared after exposure to X-ray for one, two, and three times with frequency of 19 (37.3%), 23 (45.1%), and 9 (17.6%) times, respectively, as shown in Table 1.

### 3.2. Bacterial Isolation

**Figure 1:**
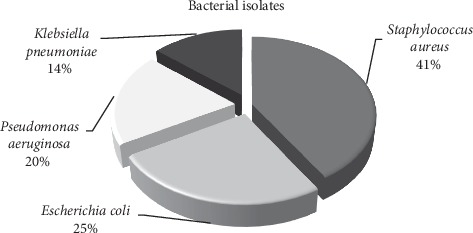
Distribution of bacterial isolates from open fractures (*n* = 51).

The bacterial isolate distribution is shown in Figure 1. According to these outcomes, *S. aureus* isolates came in the lead with 21 (41.2%) isolates among all isolated bacteria. On the other side, Gram-negative isolates formed together around 30 (59.8%) isolates and were divided as follows: *E. coli* with 13 (25.5%) isolates, *P. aeruginosa* with 10 (19.6%) isolates, and finally, *K. pneumonia*e with 7 (13.7%) isolates.

### 3.3. Antimicrobial Susceptibility Patterns

The result illustrated in Table 2 shows a high resistance level of *S. aureus* and *P. aeruginosa* isolates to most of the tested antimicrobials. *S. aureus* isolates showed high resistance patterns to oxacillin (21, 100%), clindamycin (20, 95.2%), erythromycin (19, 90.5%), cefoxitin (18, 85.7%), daptomycin (18, 85.7%), and vancomycin (16, 76.2%). A lower percentage of resistance recorded to rifampin with 5 (23.8%). Methicillin-resistant *S. aureus* (MRSA) isolated with isolation rate of 85.7% (18 isolates) depended on the resistance pattern of cefoxitin and oxacillin. All isolated *S. aureus* (21, 100%) showed a multidrug resistance (MDR) pattern. Most of MDR *S. aureus* isolates (16, 76.2%) showed resistance to six antibiotic classes.

The results of antimicrobial susceptibility test for *P. aeruginosa* isolates revealed resistance for most tested antibiotics including ceftazidime (9, 90%), cefepime (9, 90%), gentamicin (8, 80%), amikacin (8, 80%), aztreonam (7, 70%), and imipenem (7, 70%). Low level of resistance recorded against ciprofloxacin (3, 30%) and levofloxacin (3, 30%). All *P. aeruginosa* (10, 100%) isolates showed MDR pattern. Seven (70%) of *P. aeruginosa* isolates showed resistance to four antibiotic classes, as shown in Table 2.

In the study, the outcomes of susceptibility pattern for both *E. coli* and *K. pneumoniae* isolates showed resistance for all studied antibiotics except ciprofloxacin and levofloxacin which have low percentage of resistance (3 (23.1%) for *E. coli* and 2 (28.6%) for *K. pneumoniae*), as shown in Table 3. All isolated *E. coli* and *K. pneumoniae* showed resistance to ampicillin and gentamicin (13 (100%) and 7 (100%), respectively). High resistance patterns among *E. coli* isolates were reported against amoxicillin/clavulanic acid (11, 84.6%), ceftriaxone (11, 84.6%), ertapenem (10, 76.9%), trimethoprim/sulfamethoxazole (10, 76.9%), and imipenem (9, 69.3%). The resistance pattern of *K. pneumoniae* showed high resistance to amoxicillin/clavulanic acid, ceftriaxone, and trimethoprim/sulfamethoxazole with 85.7% (6 isolates) and to ertapenem and imipenem with 71.4% (5 isolates). All isolated *E. coli* (13, 100%) and *K. pneumoniae* (7, 100%) showed MDR pattern. 10 (76.9%) of *E. coli* and 5 (71.4%) of *K. pneumoniae* isolates showed resistance to 5 antibiotic classes.

### 3.4. Bacterial Isolates and Grades of Open Fractures

The relationship between bacterial isolates from preliminary culture and grades of open fractures was studied statistically using Fisher's exact test, as reported in Table 4. The results showed a statistically significant difference in the type of bacterial isolates among grades of open wound fractures (*p* < 0.001). Most of *S. aureus* isolates were isolated from grade II open fractures with isolation rate reached 95.2% (20 isolates). On the other side, Gram-negative bacteria came in the foreground among grade III classes of open fractures. The isolation rate of *P. aeruginosa* was high among grade IIIA with 6 (60%) and that of *K. pneumoniae* was higher in grade IIIB with 5 (71.4%). However, grade IIIC open wound fractures showed that the higher isolation of bacteria was recorded for *E. coli* isolates with incidence rate of 69.2% (9 isolates).

### 3.5. Number of X-Ray Exposure

An additional correlation was done between the X-ray exposure number and the type of causative bacteria to find the best X-ray exposure number for each one. The result of this comparison showed a statistically high significant relationship for all types of causative bacteria with a *p* value <0.001. The outcomes showed that the perfect time of killing *S. aureus* was one X-ray exposure with incidence rate of 85.7% (18 isolates), whereas *P. aeruginosa* and *K. pneumoniae* were dead after the second exposure of X-ray with incidence rate of 80% (8 isolates) and 100% (7 isolates), respectively. On the other side, the optimum times of X-ray exposure to *E. coli* were between two and three times of exposure with incidence rates of 46.1% (6 isolates) and 53.9% (7 isolates), respectively, as shown in Table 5.

### 3.6. Number of X-Ray Exposure and Grades of Open Fractures

In the current study, the severity of the studied fractures was arranged between grades II and III according to the Gustilo and Anderson classification. Grade II was reported in 21 (41.2%) fractures and grade III was reported in 30 (58.8%) fractures distributed on IIIA, IIIB, and IIIC with 10 (19.6%) for each one, as shown in Table 6.

Fisher's exact test was utilized to measure the relationship between the number of X-ray exposure and severity (grades) of open fractures against each type of bacteria. The results showed a statistically high significant relationship with a *p* value <0.001. Infected open fractures with grade II showed sterile culture media after the first time of X-ray exposure, and these wounds were infected with *S. aureus* or *P. aeruginosa* with 18 (90%) and 1 (100%), respectively. As for infected open fractures with the grade IIIC, these wounds showed sterile culture media after the third time of X-ray exposure, and the causative agent of wound infection was *E. coli* or *P. aeruginosa* with incidence rates of 77.8%(7 isolates) and 100% (1 isolate), respectively. On the other side, all *E. coli*, *P. aeruginosa*, and *K. pneumoniae* isolates that caused open fracture infections with grades IIIA and IIIB showed sterile culture media after the second time of X-ray exposure with an incidence rate of 100% for each type of bacteria, as mentioned in Table 6.

From the above, we concluded that Gram-positive *S. aureus* was killed after the first time of X-ray exposure, while Gram-negative bacterial isolates (*P. aeruginosa* and *K. pneumoniae*) were dead after the second time of X-ray exposure, and most *E. coli* isolates were killed after the third time of X-ray exposure.

## 4. Discussion

For several years, prevention and controlling infection for open fractures stays the crucial goal in the treatment of these wounds, so different methods were applied to achieve this goal [19, 20]. All these methods are depending on utilizing different types of antibiotic regimes [21, 22]. These antibiotic patterns benefit in several cases, but when the causative bacterial agents were multidrug resistance as shown in this study, these methods become ineffective in the treatment of open fracture infections. This problem prompted us to research other ways of treating infected open fractures.

The current study was carried out on open fractures at the lower limb because the development of infectious complexity has a greater danger at the lower limb fractures as reported by prior studies [23, 24]. Additionally, the study was conducted on open fractures arranged in severity between grades II and IIIA, B, and C, because these grades are most susceptible to develop the infection [25, 26].

The reported findings of this study demonstrated that the most common bacteria isolated from studied open fractures were *S. aureus* and Gram-negative bacteria include *E. coli*, *P. aeruginosa*, and *K. pneumonia*e. These results were identical to those recorded by Bratzler et al. in 2013 [27]. *S. aureus* recorded the highest isolation rate close to that of other studies with 48.4% and 36% of *S. aureus* isolates [28, 29]. Among isolated *S. aureus* of open fractures, the higher portion was recorded for MRSA isolates, and the high percentage of MRSA was also reported by Latha and Jain et al. with isolation rate of 57.3% and 63.29%, respectively [28, 30]. Gram-negative bacteria isolated from open fractures with isolation rate higher than the result of another study that recorded 33.34% as an isolation rate [31]. *E. coli* isolates recorded the highest isolation rate among Gram-negative bacteria, while another study showed a lower isolation rate of 13% [29]. In our study, the isolation rate of *P. aeruginosa* was close to that recorded by another study 26.3% [28]. *K. pneumoniae* isolation rate was slightly higher than the result of another study (9%) [29].

The results of antimicrobial susceptibility test showed MDR for all isolated bacterial species, as mentioned in Tables 2 and 3. In the study, the most isolated bacteria from open fractures showed resistance toward at least four classes of antibiotics, as demonstrated by Hassan et al. [17]. Most of *S. aureus* were resistant to six antibiotic classes, and this resistance pattern of *S. aureus* also reported by another study with 30.1% [32].

The results of open fractures with grade II showed infection by *S. aureus* with highest isolation rate, as was reported previously by another study [4], while open fractures with grade III showed infection with Gram-negative bacteria including *E. coli*, *P. aeruginosa*, and *K. pneumoniae* as reported by the previous study [32].

In the present study, we showed all studied infected wound fractures affected by X-ray, and this was confirmed by bacterial culture after each time of exposure. This effect of X-ray returns to the X-ray interaction with matter to produce unstable ions and free electrons. Furthermore, these free electrons may react with other atoms, which could break the DNA molecules and cause mutations. On the other side, X-ray irradiation of biological material forms reactive hydroxyl radicals, and this leads to DNA damage and other cellular macromolecules and causes cell death [33].

X-ray exposure appeared effective on *S. aureus* from the first time of exposure, while Gram-negative bacteria (*P. aeruginosa* and *K. pneumoniae*) showed effectiveness after the second time of X-ray exposure. This may return to the differences in the cell wall structure of Gram-positive and Gram-negative bacteria. The cell wall of Gram-positive bacteria has a larger amount of proteins (peptidoglycan) than that of Gram-negative bacteria [34], and X-ray has more effect on different prokaryotes by oxidative protein damage (protein carbonylation), and this leads to inactivation in the specific enzymes required in DNA repair and replication [35, 36]. In addition, X-ray radiation contains photoelectrons and Auger electrons, which damage the double-stranded DNA [37].

The controversial results of *E. coli* isolates showed that half of *E. coli* isolates were killed after the second exposure of X-ray, and the other half were killed after the third exposure; this may return to the ability of some *E. coli* isolates to develop ionizing radiation resistance by the ability to tolerate DNA damage [38].

## 5. Conclusion

In this study, we concluded that X-ray exposure shows high effectiveness in treating infected open fractures. Gram-positive *S. aureus* was the most causative agent for grade II fractures, and they were killed after the first time of X-ray exposure, while Gram-negative bacteria were the most causative agent for grade III fractures. In grade IIIA and IIIB fractures, the causative agents were killed after two times of X-ray exposure. In grade IIIC fractures, the bacterial agents were killed after three times of X-ray exposure.

## Figures and Tables

**Table 1:** Basic demographical and clinical characteristics of 51 patients suffering from open fracture.

Variables		*n* (%)
Age (years), mean ± SD		31.7 ± 5.97

Gender	Male	29 (56.9%)
	Female	22 (43.1%)

Limb fractured	Lower	51 (100%)
	Upper	0 (0%)

Infection	Yes	51 (100%)
	No	0 (0%)

Bacterial culture	Growth	51 (100%)
	No growth	0 (0%)

Times of X-ray exposure	One	19 (37.3%)
	Two	23 (45.1%)
	Three	9 (17.6%)

**Table 2:** Antimicrobial susceptibility pattern of *S. aureus* and *P. aeruginosa* isolated from infected open fractures.

*S. aureus* (*n* = 21)		*P. aeruginosa* (*n* = 10)	
Antibiotic	*n* (%)	Antibiotic	*n* (%)
Erythromycin	19 (90.5%)	Ceftazidime	9 (90%)
Oxacillin	21 (100%)	Gentamicin	8 (80%)
Cefoxitin	18 (85.7%)	Amikacin	8 (80%)
Clindamycin	20 (95.2%)	Aztreonam	7 (70%)
Daptomycin	18 (85.7%)	Cefepime	9 (90%)
Vancomycin	16 (76.2%)	Ciprofloxacin	3 (30%)
Rifampin	5 (23.8%)	Levofloxacin	3 (30%)
		Imipenem	7 (70%)

Multidrug resistance (MDR)	21 (100%)	Multidrug resistance (MDR)	10 (100%)
Resistance to 6 antibiotic classes	16 (76.2%)	Resistance to 4 antibiotic classes	7 (70%)

**Table 3:** Antimicrobial susceptibility pattern of *E. coli* and *K. pneumoniae* isolated from infected open fractures.

Antibiotic	*E. coli* (*n* = 13)	*K. pneumoniae* (*n* = 7)
	*n* (%)	*n* (%)
Ampicillin	13 (100%)	7 (100%)
Amoxicillin/clavulanic acid	11 (84.6%)	6 (85.7%)
Gentamicin	13 (100%)	7 (100%)
Ciprofloxacin	3 (23.1%)	2 (28.6%)
Levofloxacin	3 (23.1%)	2 (28.6%)
Ceftriaxone	11 (84.6%)	6 (85.7%)
Ertapenem	10 (76.9%)	5 (71.4%)
Imipenem	9 (69.3%)	5 (71.4%)
Trimethoprim/sulfamethoxazole	10 (76.9%)	6 (85.7%)

Multidrug resistance (MDR)	13 (100%)	7 (100%)
Resistance to 5 antibiotic classes	10 (76.9%)	5 (71.4%)

**Table 4:** Grades of open fractures according to the Gustilo and Anderson classification among four types of bacteria isolated by the study.

Type of bacteria	Grade of open fractures				Total *n* (%)
	II *n* (%)	IIIA *n* (%)	IIIB *n* (%)	IIIC *n* (%)	
*Staphylococcus aureus*	**20 (95.2%)**	1 (4.8%)	—	—	21 (100%)
*Pseudomonas aeruginosa*	1 (10%)	**6 (60%)**	2 (20%)	1 (10%)	10 (100%)
*Escherichia coli*	—	1 (7.7%)	3 (23.1%)	**9 (69.2%)**	13 (100%)
*Klebsiella pneumoniae*	—	2 (28.6%)	**5 (71.4%)**	—	7 (100%)

^*∗*^ *p* value = <0.001 (very high significant). ^*∗*^*p* value = Fisher's exact test with confidence interval of 99%.

**Table 5:** Times of X-ray exposure required to kill four types of isolated bacteria by the study.

Type of bacteria	Times of X-ray exposure			Total *n* (%)
	One *n* (%)	Two *n* (%)	Three *n* (%)	
*Staphylococcus aureus*	**18 (85.7%)**	2 (9.5%)	1 (4.8%)	21 (100%)
*Pseudomonas aeruginosa*	1 (10%)	**8 (80%)**	1 (10%)	10 (100%)
*Escherichia coli*	—	6 (46.1%)	**7 (53.9%)**	13 (100%)
*Klebsiella pneumoniae*	—	**7 (100%)**	—	7 (100%)

^*∗*^ *p* value = <0.001 (very high significant). ^*∗*^*p* value = Fisher's exact test with confidence interval of 99%.

**Table 6:** Times of X-ray exposure and grades of open fractures according to the type of isolated bacteria in the study.

Type of bacteria			Times of X-ray exposure			Total *n* (%)
			One *n* (%)	Two *n* (%)	Three *n* (%)	
*Staphylococcus aureus*	Grade	II	**18 (90%)**	2 (10%)	—	20 (95.2%)
		IIIA	—	—	**1 (100%)**	1 (4.8%)
	Total					**21 (100%)**
*Pseudomonas aeruginosa*	Grade	II	**1 (100%)**	—	—	1 (10%)
		IIIA	—	**6 (100%)**	—	6 (60%)
		IIIB	—	**2 (100%)**	—	2 (20%)
		IIIC	—	—	**1 (100%)**	1 (10%)
	Total					**10 (100%)**
*Escherichia coli*	Grade	IIIA	—	**1 (100%)**	—	1 (7.7%)
		IIIB	—	**3 (100%)**	—	3 (23.1%)
		IIIC	—	2 (22.2%)	**7 (77.8%)**	9 (69.2%)
	Total					**13 (100%)**
*Klebsiella pneumoniae*	Grade	IIIA	—	**2 (100%)**	—	2 (28.6%)
		IIIB	—	**5 (100%)**	—	5 (71.4%)
	Total					**7 (100%)**
Total	Grade	II	**19 (90.5%)**	2 (9.5%)	—	21 (41.2%)
		IIIA	—	**9 (90%)**	1 (10%)	10 (19.6%)
		IIIB	—	**10 (100%)**	—	10 (19.6%)
		IIIC	—	2 (20%)	**8 (80%)**	10 (19.6%)
	Total					**51 (100%)**

^*∗*^ *p* value of total = <0.05 (highly significant). ^*∗*^*p* value = Fisher's exact test with confidence intervalof 99%.

## Data Availability

The data used to support the findings of this study are included within the article.
